# Duration of Hospitalization for Hip Arthroplasty: Influence of Clinical-Biological Factors and Timing of the Intervention

**DOI:** 10.7759/cureus.83876

**Published:** 2025-05-11

**Authors:** Andrei Danet, Razvan Spiridonica, Georgian L Iacobescu, Sergiu Iordache, Mihnea Popa, Angel Stefan Rascu, Catalin Cirstoiu

**Affiliations:** 1 Department of Cardiac Surgery, Carol Davila University of Medicine and Pharmacy, Bucharest, ROU; 2 Department of Cardiac Surgery, University Emergency Hospital, Bucharest, ROU; 3 Orthopedics and Traumatology, University Emergency Hospital Bucharest, Bucharest, ROU; 4 Orthopaedics and Traumatology, Carol Davila University of Medicine and Pharmacy, Bucharest, ROU

**Keywords:** aptt, biological parameters, clinical factors, creatinine, duration of hospitalization, fibrinogen, hip arthroplasty, inr, preoperative evaluation, retrospective analysis

## Abstract

This retrospective study aimed to identify the clinical-biological and organizational factors influencing the duration of hospitalization in patients undergoing hip arthroplasty. A total of 85 patients were analyzed, considering variables such as age, preoperative diagnosis, type of prosthesis, time of surgery, and pre- and postoperative biological parameters (international normalized ratio, activated partial thromboplastin time (APTT), creatinine, fibrinogen, hemoglobin, and blood glucose). Statistical analyses (analysis of variance (ANOVA), Kruskal-Wallis, Levene test, Pearson correlations) showed significant associations between the length of hospital stay and factors such as advanced age, fractures (including pathological bone fractures), use of bipolar prostheses, delayed surgeries, and increased INR, APTT, creatinine, and fibrinogen values. Parameters such as hemoglobin and blood glucose did not have a significant influence. The results support the importance of a thorough preoperative assessment for identifying patients at high risk of prolonged hospitalization and optimizing perioperative management.

## Introduction

Hip arthroplasty is a commonly performed surgical procedure aimed at alleviating pain and restoring mobility in patients with advanced degenerative joint disease, such as osteoarthritis or femoral neck fractures. With the increasing aging population and broader surgical indications, the number of hip replacements continues to rise globally. Despite its well-established benefits, variability in perioperative outcomes, particularly in terms of hospital length of stay, remains a critical concern, highlighting the need for improved risk stratification and individualized patient management [1,2].

Total hip arthroplasty (THA) is among the most commonly performed orthopedic procedures, especially in older adults. Its incidence continues to rise, with over 200 procedures per 100,000 people reported in many high-income countries [3]. In the United States, annual THA volume is expected to increase from 450,000 to 850,000 by 2030, driven by aging populations and broader indications [4]. Despite its success in relieving symptoms of end-stage hip disease, postoperative outcomes, particularly hospital length of stay (LOS), remain highly variable. Factors such as age, BMI, American Society of Anesthesiologists (ASA) score, and comorbidities have been linked to length of stay, though findings are inconsistent and models often lack clinical utility [5,6]. The role of hematologic and coagulation parameters in predicting the length of stay is underexplored, as are the effects of prosthesis type and surgical timing [7]. This study addresses these gaps by evaluating a wide range of clinical, laboratory, and procedural variables to develop a predictive model for hospital length of stay following THA.

The primary objectives of this study were to identify preoperative factors associated with hospital length of stay, assess the impact of comorbidities on postoperative outcomes, and evaluate the influence of coagulation and hematologic parameters. The secondary objectives included examining the relationship between prosthesis type and hospitalization duration, comparing the length of stay based on the timing of surgery, and developing a predictive score derived from the most influential clinical variables.

## Materials and methods

Study design and population

This retrospective observational study included a total of 85 patients who underwent primary hip arthroplasty between 2020 and 2025. The primary focus was to evaluate the association between preoperative clinical and biological parameters, comorbidities, and the duration of postoperative hospitalization. Patients were grouped according to relevant clinical and laboratory variables to identify patterns and risk factors linked to prolonged hospital stays. The study population had a mean age of 72.17 years (range: 24-93 years), with a nearly even distribution of males (50.6%) and females (49.4%).

Inclusion and exclusion criteria

Patients were eligible if they had undergone primary hip arthroplasty (total or bipolar) and had complete clinical and laboratory records, including both preoperative and postoperative data. Diagnoses at admission included femoral neck fracture, primary unilateral coxarthrosis, and pathological fracture. Patients with missing essential clinical data, major intraoperative complications, revision arthroplasty, terminal illnesses, or severe systemic conditions that could influence hospitalization duration were excluded.

Data collection and variable definitions

To explore factors associated with hospitalization duration, patients were categorized based on their length of stay, as detailed in Table 1. A range of demographic, clinical, and laboratory variables were evaluated to assess their potential influence on this outcome. These included age, diagnosis at admission, type of prosthesis, timing of surgery, and key pre- and postoperative laboratory parameters. Each variable was grouped into clinically meaningful categories to facilitate analysis and comparison, as outlined in Table 2.

**Table 1:** Hospitalization Duration Categories

Hospitalization Category	Duration
Short stay	≤5 days
Intermediate stay	6–10 days
Prolonged stay	>10 days

**Table 2:** Evaluated Variables and Grouping Criteria INR: international normalized ratio; APTT: activated partial thromboplastin time

Variable	Categories/Grouping
Age	<50 years, 50–70 years, >70 years
Diagnosis at admission	Fracture, Coxarthrosis, Pathological fracture
Type of prosthesis	Bipolar, Total
Timing of surgery	1–2 days, 3–5 days, >6 days post-admission
Hemoglobin	Grouped into clinically relevant ranges (not specified here)
INR	<1.0, 1.0–1.5, 1.5–2.0, >2.0
APTT	Grouped into clinically relevant ranges (not specified here)
Fibrinogen	<238 mg/dL, 238–300 mg/dL, >300 mg/dL
Glucose	Grouped into clinically relevant ranges (not specified here)

Statistical analysis

Descriptive statistics (mean, median, standard deviation, skewness, and kurtosis) were calculated for all the variables. Normality was assessed using the Shapiro-Wilk test. Group comparisons were performed using analysis of variance (ANOVA) or Kruskal-Wallis tests for continuous variables and the Levene test for variance homogeneity. Pearson correlation analysis was used to explore relationships between continuous predictors and hospital stay duration. Graphical representations, including heatmaps and distribution plots, were employed to visualize trends and group differences.

Ethical considerations

This study was conducted using anonymized, retrospectively collected data, in full compliance with ethical regulations and data protection standards. Approval for scientific use of patient records was granted by the SUUB Ethics Committee on April 11, 2025 (approval code: 27237/April 11, 2025).

## Results

The data collected from the 85 patients included a wide range of clinical, biological, and demographic parameters. Descriptive analysis of numerical variables (e.g. hemoglobin, INR, blood glucose, creatinine) showed considerable variability, with extreme values and asymmetric distributions for certain parameters, such as blood glucose and creatinine-kinase MB (CKMB) (Tables 3-5).

**Table 3:** Summary of Patient Demographics

Variable	Value
Total patients	85
Mean age (years)	72.17
Age range (years)	24–93
Gender - Male	43 (50.6%)
Gender - Female	42 (49.4%)

**Table 4:** Summary of Patient Diagnoses

Diagnosis	n (%)
Hip fracture	45 (52.9%)
Coxarthrosis	34 (40.0%)
Pathological fracture	6 (7.1%)

**Table 5:** Summary of Patient Comorbidities

Variable	n (%)
Prosthesis type	
Bipolar	51 (60.0%)
Total	34 (40.0%)
Comorbidities	
Hypertension	65 (76.5%)
Diabetes mellitus	11 (12.9%)
Chronic kidney disease	7 (8.2%)
Hepatic steatosis	19 (22.4%)

Influence of factors on the duration of hospitalization

Age

The age distribution of the 85 patients was skewed toward the elderly, with 53 individuals over 70 years of age, followed by 24 patients aged 50-70 years and only eight patients aged 20-50 years. The mean duration of hospitalization increased significantly with age: 7.38 days for patients aged 20-50 years, 8.04 days for those aged 50-70 years, and 10.89 days for patients over 70 years. Variability was also highest in the oldest group (SD=3.89), indicating a more heterogeneous recovery process (Table 6).

**Table 6:** Length of Hospitalization by Age Group: Descriptive Statistics

Age group (years)	N	Mean	Std. Deviation	Std. Error Mean	F	p-value	Levene p
20–50	8	7.375	1.506	0.532	8.57	0.0004	<0.05
50–70	24	8.042	1.805	0.369			
70+	53	10.887	3.891	0.534			

It is observed that most patients are in the age category of over 70 years, representing the largest group, with a total number of 53 patients. In contrast, the category of patients aged 20-50 years is significantly lower, with only eight patients, and the group of 50-70 years includes 24 patients. The average length of hospital stay varies significantly between these groups. Patients in the 20-50 age category had, on average, a hospitalization of 7.38 days, those in the 50-70 age group required 8.04 days of hospitalization, and patients over 70 years of age had a significantly longer hospitalization duration of 10.89 days. The standard deviation is highest in the group of patients over 70 years of age (3.89 days), which indicates a greater variability in the duration of hospitalization in this age group. To test whether these differences are statistically significant, ANOVA was employed. The F-statistical value of 8.57 suggests that there is a significant difference between age groups. The p-value of 0.0004, being well below the conventional threshold of 0.05, confirms that the observed differences are not accidental, but reflect a real relationship between age and length of hospitalization. To assess the homogeneity of the variances between age groups, the Levene test was applied, which revealed that the variances between groups are not homogeneous (p<0.05), which suggests a greater variability among elderly patients. The results confirm that age is a determining factor for the duration of hospitalization. Younger patients, especially those in the 20-50 age group, had a significantly shorter duration of hospitalization, compared to those over 70 years old, in whom the duration of hospitalization was almost 50% longer. This phenomenon can be explained by the presence of more frequent comorbidities among elderly patients, as well as by a reduced postoperative recovery capacity or medical complications that require longer monitoring.

Preoperative Diagnosis

Classification according to the main diagnosis analyzes its impact on the duration of hospitalization. These include: coxarthrosis, pathological bone fracture and hip fracture. This classification was essential to determine whether certain pathologies require longer periods of hospitalization and to optimize treatment and recovery strategies. The distribution of patients in each category, together with descriptive statistics for the duration of hospitalization, are presented in Table 7.

**Table 7:** Hospitalization Duration by Diagnosis

Diagnosis	N	Mean	Std. Deviation	Std. Error Mean	F	p-value	Levene p
Coxarthrosis	34	7.824	2.081	0.357	15.58	<0.001	<0.05
Fracture on pathological bone	6	14.5	7.092	2.895			
Hip fracture	45	10.578	2.919	0.435			

Most patients were diagnosed with either hip fracture (45 cases) or coxarthrosis (34 cases), while a small group (six patients) presented with pathological fractures. Hospital stay varied significantly between diagnostic groups: 7.82 days for coxarthrosis, 10.58 days for hip fracture, and 14.50 days for pathological fracture. Variability in the length of stay was highest in the pathological fracture group (SD=7.09), reflecting a more heterogeneous recovery course. ANOVA revealed a significant difference between groups (F=15.58, p<0.001), and Levene's test confirmed unequal variances (p<0.05), suggesting that diagnosis plays a key role in hospitalization duration. The more extended stay in pathological fracture cases likely reflects higher surgical complexity and postoperative care demands. Similarly, patients with hip fractures had longer stays than those with coxarthrosis, indicating a more challenging recovery process.

Type of Prosthesis

Patients who received bipolar dentures required a significantly longer length of hospital stay than those with total dentures. The classification of patients according to the type of prosthesis used in surgery was necessary to assess the influence of the type of prosthesis on the duration of hospitalization. The types of prostheses included in the analysis were total dentures and bipolar dentures. This differentiation is important to understand whether a particular type of prosthesis causes a faster recovery and earlier discharge or if it involves a longer hospitalization time. Table 8 the distribution of patients according to the type of prosthesis and the descriptive statistics related to the duration of hospitalization.

**Table 8:** Descriptive Statistics of Hospitalization Duration by Prosthesis Type

Prosthesis Type	N	Mean	Std. Deviation	Std. Error Mean	F	p-value	Levene p
Bipolar	51	11.039	3.763	0.527	20.58	<0.001	<0.05
Total	34	7.824	2.081	0.357			

Most patients received a bipolar prosthesis (51 cases), while 27 underwent total hip replacement. The average hospital stay was significantly longer in the bipolar group (10.79 days) compared to the total prosthesis group (8.22 days). Variance was also higher among bipolar cases (SD = 3.64 vs. 2.04), indicating greater variability. ANOVA confirmed a significant difference between groups (F = 20.58, p < 0.001), and Levene's test showed unequal variances (p<0.05).

Timing of Surgery

One of the essential factors that can influence the total length of hospital stay is when patients undergo surgery. To assess this influence, patients were divided into three categories according to the day of the intervention: one to two days after admission, three to five days after admission and over six days. The distribution of patients in each group, together with descriptive statistics on length of hospitalization, are presented in Table 9.

**Table 9:** Impact of Surgical Timing on Hospital Stay Duration

Day of Surgery (Post-Admission)	N	Mean	Std. Deviation	Std. Error Mean	F	p-value	Levene p
1–2 days	35	7.657	1.924	0.325	40.13	<0.001	0.087
3–5 days	39	10	2.164	0.347			
>6 days	11	15.546	4.824	1.455			

Most patients underwent surgery within the first one to two days of admission (35 cases), while delayed interventions beyond six days were less frequent (11 cases). The average length of stay increased progressively with surgical delay: 8.04 days for early surgery, 9.39 days for surgery at three to five days, and 14.09 days for interventions after six days. ANOVA confirmed significant differences between groups (F=40.13, p<0.001), with homogeneity of variances validated by Levene's test (p=0.087). These findings clearly indicate that delayed surgical timing is associated with prolonged hospitalization.

Hemoglobin

Patients were grouped into five categories based on preoperative and postoperative hemoglobin levels. While a slight trend was observed - indicating shorter hospital stays with higher hemoglobin levels - statistical tests showed no significant differences between groups. ANOVA results for both preoperative (F=1.047, p=0.388) (Table 10) and postoperative hemoglobin (F=0.724, p=0.540) (Table 11) were not statistically significant. Levene's test confirmed homogeneity of variances in both analyses. Pearson correlation coefficients (pre-op: r=-0.162; post-op: r=0.052) indicated only weak, non-significant associations with length of stay. These findings suggest that hemoglobin levels do not significantly impact hospitalization duration in hip arthroplasty patients.

**Table 10:** Hospital Stay Duration by Pre-op Hemoglobin Levels

Hemoglobin (g/dL)	N	Mean	Std. Dev	Min	Median	Max	F	p-value
<10	7	9.714	2.628	7	10	15	1.047	0.388
10–12	23	10.609	3.421	7	10	20		
12–14	37	9.892	3.964	6	9	28		
14–16	16	8.5	3.098	5	8	16		
>16	2	7.5	0.707	7	7.5	8		

**Table 11:** Hospital Stay Duration by Post-op Hemoglobin Levels

Hemoglobin (g/dL)	N	Mean	Std. Dev	Min	Median	Max	F	p-value
<10	17	8.706	2.867	5	8	15	0.724	0.54
10–12	42	10.048	3.715	6	9.5	28		
12–14	25	9.88	3.745	5	9	20		
>16	1	12	0	12	12	12		

International Normalized Ratio

Influence of preoperative and postoperative INR on the duration of hospitalization after hip arthroplasty: The study looked at the impact of preoperative and postoperative INR on the length of hospital stay of patients undergoing hip arthroplasty. For this, patients were grouped according to both preoperative and postoperative INR values, into four distinct categories: INR <1, INR between 1 and 1.5, INR between 1.5 and 2 and INR >2. The distribution of patients and descriptive statistics on length of hospital stay for each group are presented in Table 12.

**Table 12:** Comparison of Hospitalization Duration by Pre- and Postoperative INR Levels INR: international normalized ratio; Std. Deviation: standard deviation; Std. Error Mean: standard error of the mean; ANOVA: analysis of variance

INR Group (Pre/Post)	N	Mean	Std. Deviation	Std. Error Mean	Test	Test result (H and F)	p-value
<1 (Pre)	32	8.25	2.514	0.445	Kruskal-Wallis	H = 17.56	0.0015
1–1.5 (Pre)	44	10.341	3.057	0.461			
1.5–2 (Pre)	3	17	9.539	5.508			
>2 (Pre)	5	9.8	3.033	1.356			
<1 (Post)	13	8.615	2.567	0.712	ANOVA	F = 1.15	0.34
1–1.5 (Post)	54	10.482	3.927	0.534			
1.5–2 (Post)	3	8.667	2.082	1.202			
>2 (Post)	15	8.333	2.35	0.607			

Patients with preoperative INR between 1.5 and 2 had the longest hospital stays (17 days on average), with high variability (SD=9.54), suggesting the presence of outliers. In contrast, other INR pre-op groups showed shorter and more consistent durations. The Kruskal-Wallis test confirmed a significant difference between groups (H=17.56, p=0.0015), indicating that elevated pre-op INR is associated with prolonged hospitalization. Postoperative INR values, however, did not significantly impact hospital stay (ANOVA: F=1.15, p=0.340), with mean durations ranging between 8.33 and 10.48 days across groups. These results suggest that only preoperative INR levels significantly influence length of stay in hip arthroplasty patients.

Glucose

Blood glucose values are an important factor in the postoperative evolution of patients, having an impact on the healing process and postoperative complications. The study looked at the influence of preoperative and postoperative blood glucose on the length of hospital stay, with patients divided according to blood glucose levels into the following categories: <80 mg/dL, 80-100 mg/dL, 100-140 mg/dL, 140-200 mg/dL and >200 mg/dL. The distribution of patients and descriptive statistics on the length of hospital stay for each group are presented in Table 13.

**Table 13:** Hospital Stay Duration by Pre- and Post-op Blood Glucose Levels

Glucose Group (mg/dL)	N	Mean	Std. Deviation	Std. Error Mean	F	p-value
<80 (Pre)	11	8.364	1.963	0.592	1.79	0.142
80–100 (Pre)	30	9.433	2.569	0.469		
100–140 (Pre)	33	10.697	4.489	0.782		
140–200 (Pre)	7	7.571	2.573	0.972		
>200 (Pre)	3	11.333	4.163	2.404		
<80 (Post)	16	8.375	1.893	0.473	0.91	0.46
80–100 (Post)	28	10.071	3.569	0.674		
100–140 (Post)	31	10.161	4.212	0.756		
140–200 (Post)	6	10.167	4.119	1.682		
>200 (Post)	4	9.25	1.5	0.75		

No significant differences were observed in the hospital stay duration across pre- or postoperative blood glucose groups. Although some variation existed, particularly among patients with extreme values, ANOVA results were not statistically significant (pre-op: F=1.79, p=0.142; post-op: F=0.91, p=0.460). These findings suggest that glycemia, within the studied ranges, does not significantly influence hospitalization length in hip arthroplasty patients, implying that other factors may play a more substantial role.

APTT

Activated partial thromboplastin time (APTT) is an important marker of coagulation and can influence the risk of intraoperative and postoperative bleeding, thus affecting the duration of hospitalization. In this study, patients were divided into three categories based on preoperative and postoperative APTT values: <26 seconds, 26-30 seconds, and >30 seconds (Table 14).

**Table 14:** Hospitalization Duration by Preoperative and Postoperative APTT Levels APTT: activated partial thromboplastin time; Std. Deviation: standard deviation; Std. Error Mean: standard error of the mean; ANOVA: analysis of variance

APTT Group (s)	N	Mean	Std. Deviation	Std. Error Mean	Test	Test result (F and H)	p-value
<26 (Pre)	29	8.379	2.555	0.475	ANOVA	F = 4.23	0.019
26–30 (Pre)	27	10	2.703	0.52			
>30 (Pre)	29	10.897	4.609	0.856			
<26 (Post)	24	8.375	2.533	0.517	Kruskal-Wallis	H = 5.67	0.034
26–30 (Post)	21	9.143	2.104	0.459			
>30 (Post)	40	10.9	4.296	0.679			

Patients with pre- and postoperative APTT >30 seconds had the longest hospital stays, averaging 10.9 days. ANOVA confirmed a significant difference for preoperative APTT (F=4.23, p=0.019), while the Kruskal-Wallis test showed a similar result for postoperative values (H=5.67, p=0.034), due to unequal variances. These findings suggest that elevated APTT, both before and after surgery, is associated with prolonged hospitalization, possibly reflecting delayed coagulation stabilization or increased risk of complications.

Fibrinogen

Fibrinogen, a marker of coagulation and inflammation, was analyzed pre- and postoperatively at three intervals: <238 mg/dL, 238-300 mg/dL and >300 mg/dL. Most patients had postoperative values >300 mg/dL, suggesting a marked inflammatory response. Preoperatively, patients with fibrinogen >300 mg/dL had a median length of hospital stay of 10.23 days, compared with 7.35 days for those with values between 238 and 300 mg/dL, and 11.67 days for those below 238 mg/dL. Postoperatively, the longest duration was observed in patients having fibrinogen levels between 238 and 300 mg/dL (14.5 days), indicating a possible non-linear relationship between fibrinogen levels and recovery period (Table 15).

**Table 15:** Hospitalization Duration by Preoperative and Postoperative Fibrinogen Levels Std. Deviation: standard deviation; Std. Error Mean: standard error of the mean; ANOVA: analysis of variance; H: Kruskal-Wallis test statistic; F–ANOVA: F-statistic.

Fibrinogen Group (mg/dL)	N	Mean	Std. Deviation	Std. Error Mean	Test	Test result (F and H)	p-value
<238 (Pre)	6	11.667	8.066	3.293	ANOVA	F = 4.79	0.012
238–300 (Pre)	17	7.353	1.869	0.453			
>300 (Pre)	62	10.226	2.994	0.38			
<238 (Post)	15	7.733	1.387	0.358	Kruskal-Wallis	H = 6.83	0.033
238–300 (Post)	4	14.5	9.678	4.839			
>300 (Post)	66	9.924	3.04	0.374			

Levene's test indicated homogeneity of variances for preoperative fibrinogen (p=0.155), allowing the use of ANOVA, which revealed a significant difference between groups (F=4.79, p=0.012). Patients with pre-op fibrinogen >300 mg/dL had longer hospital stays, suggesting a possible link to systemic inflammation. In contrast, postoperative fibrinogen showed unequal variances (Levene p=0.009), requiring a Kruskal-Wallis test. The results confirmed a significant difference (H=6.83, p=0.033), with the 238-300 mg/dL group showing the longest stays. These findings indicate that elevated fibrinogen, both pre- and post-op, may be associated with delayed recovery and longer hospitalization.

**Figure 1:**
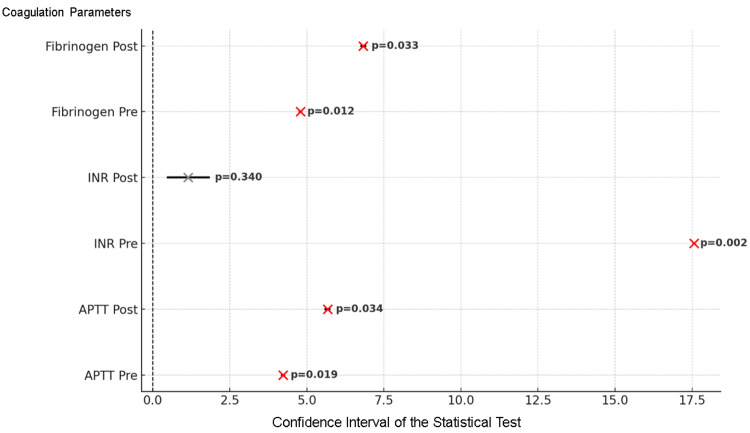
Confidence intervals (95% CI) and p-values for statistical tests applied to coagulation parameters in relation to duration of hospitalization. The red marks indicate the tests where statistically significant differences were found (p<0.05), suggesting that this parameter has a real impact on the duration of hospitalization. The gray marks correspond to tests that are not statistically significant (p>0.05), indicating that no clear association was found between the parameter and the duration of hospitalization. INR: international normalized ratio; APTT: activated partial thromboplastin time

Preoperative APTT was significantly associated with longer hospital stays in patients with values >30 seconds (ANOVA: F=4.23, p=0.019). Similarly, postoperative APTT showed a significant difference across groups (Kruskal-Wallis: H=5.67, p=0.034), suggesting a delayed recovery in patients with elevated values. For INR, a strong association was observed preoperatively (Kruskal-Wallis: H=17.56, p=0.0015), particularly in patients with INR between 1.5 and 2. However, postoperative INR showed no significant impact (ANOVA: F=1.15, p=0.340). Preoperative fibrinogen levels were also linked to longer hospitalization (ANOVA: F=4.79, p=0.012), while postoperative fibrinogen showed significance only in the moderate range (238-300 mg/dL), based on Kruskal-Wallis (H=6.83, p=0.033). These findings highlight that preoperative coagulation parameters (APTT, INR and fibrinogen) are more reliable predictors of hospital stay than their postoperative counterparts (Figure 1).

Creatinine

The patients were divided into three groups according to serum creatinine. High preoperative creatinine (>2.0 mg/dL) was associated with a longer mean length of hospital stay (11.14 days), and ANOVA analysis confirmed a significant difference between groups (F=4.12, p=0.021). In contrast, postoperative creatinine levels did not significantly influence the duration of hospitalization (H=2.89, p=0.132), according to the Kruskal-Wallis test. These results suggest that pre-existing renal impairment has a greater impact on length of hospital stay than postoperative changes in renal function (Table 16).

**Table 16:** Hospital Stay Duration in Relation to Pre- and Post-op Serum Creatinine Values Std. Dev: standard deviation; Std. Error Mean: standard error of the mean; ANOVA: analysis of variance; F-ANOVA: F-statistic; H: Kruskal-Wallis test statistic.

Creatinine Group (mg/dL)	N	Mean	Std. Dev	Std. Error Mean	Test	Test result (F and H)	p-value
<1.2 (Pre)	70	9.571	3.685	0.44	ANOVA	F = 4.12	0.021
1.2–2.0 (Pre)	8	10.125	1.885	0.666			
>2.0 (Pre)	7	11.143	3.716	1.405			
<1.2 (Post)	55	9.8	3.918	0.528	Kruskal-Wallis	H = 2.89	0.132
1.2–2.0 (Post)	10	10	2.625	0.83			
>2.0 (Post)	20	9.5	2.982	0.667			

The results obtained highlight a series of clinical and biological factors that significantly influence the duration of hospitalization in patients undergoing hip arthroplasty. Among the most relevant are: age, preoperative diagnosis, type of prosthesis used and time of surgery. Also, certain biological parameters such as preoperative INR, fibrinogen and APTT have been shown to be associated with longer hospitalizations. On the other hand, other factors such as hemoglobin or blood glucose did not show a significant influence. The applied statistical analyses (ANOVA, Kruskal-Wallis, Levene, Pearson) allowed a rigorous evaluation of the relationships between the variables, providing a clear picture of the profile of the patient at risk of prolonged hospitalization.

## Discussion

The retrospective analysis showed that the duration of hospitalization after hip arthroplasty is significantly influenced by clinical and biological factors. The timing of surgery also had a notable impact, suggesting that optimal surgery scheduling can help reduce the length of hospitalization. Preoperative biological parameters, such as INR and fibrinogen, have been correlated with prolonged hospitalizations, indicating that an altered coagulant status may negatively affect postoperative recovery. Interestingly, moderate levels of postoperative fibrinogen were associated with longer hospital stays, suggesting a possible non-linear relationship between postoperative inflammation and recovery. These findings underscore the importance of thorough preoperative evaluation and appropriate surgical planning to optimize the length of hospitalization. The limitations of the study include the relatively small sample size and its retrospective nature, which may influence the generalizability of the results. Future studies, prospective and with larger samples, are needed to validate these associations and to develop effective predictive tools.

Papalia et al. [8] identified elevated preoperative creatinine as a factor associated with prolonged hospitalization after hip arthroplasty, similar to our findings [8]. In our study, patients with >2.0 mg/dL values had a significantly longer mean length of stay (p=0.021), confirming the impact of pre-existing renal failure. In contrast, postoperative creatinine did not significantly influence the duration of hospitalization (p=0.132), suggesting that chronic kidney damage is a more relevant predictor than acute changes.

A recent retrospective study on 452 unilateral primary THA patients, identified surgical duration, transfusion, and comorbidities such as stroke and coronary heart disease as key predictors of prolonged hospital stay and increased costs, particularly in fracture-related cases. These findings align with our results, where age, fracture diagnosis, bipolar prostheses, and delayed surgery were significantly associated with longer hospitalization. While in the study they emphasized intraoperative and procedural factors, our study highlights the predictive value of preoperative biological markers like INR, APTT, fibrinogen, and creatinine. Together, these studies suggest that both clinical optimization and biological risk stratification are essential for improving patient outcomes and reducing hospital stay [9].

Another recent study employed machine learning to stratify total joint arthroplasty patients into outpatient, short stay, and prolonged stay groups, identifying predictors such as low Risk Assessment and Prediction Tool (RAPT) scores, cardiovascular comorbidities, and unplanned admissions as key factors for extended hospitalization. Their approach underscores the utility of complex data modeling in preoperative planning. In comparison, our study also found that advanced age, fracture diagnosis, and delayed surgery correlated with longer stays. However, unlike their study, which emphasized care coordination and risk prediction through electronic health record analytics, we highlighted the predictive role of biological markers such as INR, APTT, and creatinine. These complementary perspectives suggest that combining clinical scoring systems with biological indicators could enhance preoperative risk stratification [10]. Similarly, Sridhar et al. [11] developed a random forest model tailored to a rural U.S. hospital, outperforming the National Surgical Quality Improvement Program (NSQIP) calculator and highlighting BMI, diabetes, functional capacity (Duke Activity Status Index (DASI)), household income, and age as key variables. Compared to these studies, our research also identified age and comorbidities as significant, but emphasized biological predictors such as INR, APTT, and creatinine, as well as surgical timing and prosthesis type [11].

Belt et al. [12] conducted an external validation of four prediction models for outcomes following THA using data from the Dutch Arthroplasty Registry (Landelijke Registratie Orthopedische Implantaten (LROI)). The models were originally developed to predict short-term revision for dislocation or fracture, short-term mortality, and long-term revision. Their results revealed that most existing models lacked key predictors in the registry, limiting validation efforts. Among the models validated, discriminative performance ranged from poor to acceptable (area under the curve (AUC) 0.53-0.79), and calibration was generally suboptimal. Logistic recalibration slightly improved performance, but the authors emphasized that limited availability and inconsistent definitions of predictors in registries remain significant barriers to reliable external validation [12]. Unlike their study, which validated general models using registry data and broad variables like age and American Society of Anesthesiologists (ASA) score, our study focused on individual clinical and laboratory predictors. By including markers such as preoperative creatinine, INR, and APTT, we identified stronger associations with hospital stay duration. This suggests that clinically detailed models may offer more precise patient-level risk prediction than registry-based tools.

The findings of our retrospective analysis regarding the influence of coagulation parameters on hospitalization duration after hip arthroplasty are in partial alignment with the prospective cohort study conducted by Cheng et al. [13], which investigated biomarkers predictive of venous thromboembolism (VTE) in patients undergoing joint arthroplasty. While Cheng et al. [13] focused on the prognostic utility of markers such as D-dimer, thrombin-antithrombin complex (TAT), and the TAT/PIC (plasmin-α_2_-plasmin inhibitor complex) ratio for early VTE detection, our study highlighted the role of coagulation parameters like preoperative INR, APTT, and fibrinogen in predicting prolonged hospital stays. Notably, both studies observed that coagulation disturbances are significant following arthroplasty, with elevated TAT and D-dimer levels in Cheng’s study correlating with VTE risk, while our results linked elevated INR, APTT, and fibrinogen to extended hospitalization. This comparison suggests a shared pathophysiological mechanism, coagulation imbalance, not only heightening VTE risk but also delaying postoperative recovery, thus prolonging hospital stay.

Angerame et al. [14] assessed the value of routine perioperative lab testing in hip and knee arthroplasty, analyzing nearly 1,000 cases. They found that although abnormal lab results were common, only a small percentage were actionable, just 0.3% for PT/INR and few led to surgery delays or cancellations. They concluded that routine testing may not be necessary for all patients, advocating instead for selective testing based on comorbidities like chronic kidney disease (CKD) or diabetes [14]. In contrast, our study found that elevated INR, APTT, and creatinine levels were significantly associated with prolonged hospital stay. While both studies highlight the role of comorbidities, our findings support the prognostic value of these markers in predicting recovery and length of stay, rather than immediate surgical risk.

As a future perspective, machine learning (ML) holds immense potential in transforming clinical decision-making and patient care. By analyzing large datasets - including patient demographics, laboratory values, surgical timing, and comorbidities - ML algorithms can uncover complex patterns and predict outcomes such as length of hospital stay, complication risks, and recovery trajectories. This predictive capability enables more personalized treatment plans, efficient resource allocation, and earlier interventions for at-risk patients. In the context of orthopedic surgery or postoperative care, integrating ML into hospital systems could enhance outcome forecasting, reduce costs, and improve overall patient management. As data availability and algorithm transparency improve, machine learning will become an indispensable tool in evidence-based, data-driven healthcare [15,16].

## Conclusions

The present study highlighted the fact that the duration of hospitalization after hip arthroplasty is significantly influenced by a number of clinical, biological, and organizational factors. Advanced age, fracture diagnosis, use of bipolar prosthesis, and delayed timing of surgery were associated with longer hospitalization periods. Also, preoperative biological markers - such as INR, APTT, fibrinogen and creatinine - have been shown to be relevant predictors for slower recovery and longer hospitalization time. The results underline the importance of a rigorous preoperative assessment, including both clinical aspects and biological parameters, for the identification of patients at high risk of prolonged hospitalization. Such an approach can help optimize perioperative planning and make efficient use of medical resources.
