# Correction: Duration of Hospitalization for Hip Arthroplasty: Influence of Clinical-Biological Factors and Timing of the Intervention

**DOI:** 10.7759/cureus.c283

**Published:** 2025-09-05

**Authors:** Andrei Danet, Razvan Spiridonica, Georgian L Iacobescu, Sergiu Iordache, Mihnea Popa, Angel Stefan Rascu, Catalin Cirstoiu

**Affiliations:** 1 Department of Cardiac Surgery, Carol Davila University of Medicine and Pharmacy, Bucharest, ROU; 2 Department of Cardiac Surgery, University Emergency Hospital, Bucharest, ROU; 3 Orthopedics and Traumatology, University Emergency Hospital Bucharest, Bucharest, ROU; 4 Orthopaedics and Traumatology, Carol Davila University of Medicine and Pharmacy, Bucharest, ROU

This article has been revised to add an affiliation for Dr. Andrei Danet. His first affiliation is: Department of Cardiac Surgery, Carol Davila University of Medicine and Pharmacy, Bucharest, Romania.

